# Intra-Laboratory Validation of Alpha-Galactosidase Activity Measurement in Dietary Supplements

**DOI:** 10.3390/molecules26061566

**Published:** 2021-03-12

**Authors:** Elena Fabris, Michela Bulfoni, Alessandro Nencioni, Emanuele Nencioni

**Affiliations:** 1 Biofarma Group Srl, Via Castelliere 2, Mereto di Tomba, 33036 Udine, Italy; cqaccettazione@biofarma.it; 2 Institute of Pathology Department of Medicine, University of Udine, 33100 Udine, Italy; michela.bulfoni@uniud.it; 3 IBSA Institut Biochimique SA, Via del Piano29, CH-6915 Pambio Noranco, Switzerland; Alessandro.Nencioni@ibsa.ch

**Keywords:** dietary supplements, alpha-galactosidase, quality control, spectrophotometric assay, method validation

## Abstract

Introduction: Alpha-galactosidase (α-Gal) is an enzyme responsible for the hydrolyzation of glycolipids and glycoprotein commonly found in dietary sources. More than 20% of the general population suffers from abdominal pain or discomfort caused by intestinal gas and by indigested or partially digested food residuals. Therefore, α-Gal is used in dietary supplements to reduce intestinal gases and help complex food digestion. Marketed enzyme-containing dietary supplements must be produced in accordance with the Food and Drug Administration (FDA) regulations for Current Good Manufacturing Practice (cGMPs). Aim: in this work we illustrated the process used to develop and validate a spectrophotometric enzymatic assay for α-Gal activity quantification in dietary supplements. Methods: The validation workflow included an initial statistical-phase optimization of materials, reagents, and conditions, and subsequently a comparative study with another fluorimetric assay. A final validation of method performance in terms of specificity, linearity, accuracy, intermediate-precision repeatability, and system precision was then executed. Results and conclusions: The proven method achieved good performance in the quantitative determination of α-Gal activity in commercial food supplements in accordance with the International Council for Harmonisation of Technical Requirements for Pharmaceuticals (ICH) guidelines and is suitable as a rapid in-house quality control test.

## 1. Introduction

Alpha-galactosidase (α-Gal) is an enzyme expressed in the mammal tissues responsible for hydrolyzing terminal alpha galactosyl moieties from glycolipids and glycoproteins commonly found in dietary sources [1,2]. In particular, α-galactosidase is able to hydrolyze galacto-oligosaccharides such as starch, raffinose, melibiose and branched polysaccharides, galactomannans, and galacto-glucomannans, which catalyze the hydrolysis of α-1,6 linked galactose residues [1,2,3,4]. This enzyme is produced in the mouth in the form of saliva, as well as in the pancreas from where it is moved into the small intestine and the rest of the digestive tract in order to perform its function [2,4]. If α-galalactosidase production is insufficient due to age, genetics, or for any other reason, the chances of having undigested or partially digested food particles in our digestive tracts, as well as abdominal cramps, gas, and yeast infections, increase [2,5,6,7].

An adequate exogenous supply of α-galactosidase, through drugs or supplements, allows for the breakdown of complex carbohydrates into fructose, glucose, and galactose before they reach the colon. Therefore, α-galactosidase prevents these carbohydrates from becoming an anaerobic fermentation substrate [7,8,9,10,11]. To improve the nutritional value of products and make them easily digestible, α-galactosidases are often supplemented in food [12,13,14]. These enzymes must resist various gut proteases in order to function properly in the human gut [2,4,6,7,15]. Highly efficient α-galactosidases with protease resistance are urgently needed for this reason, among others. However, only a few protease-resistant α-galactosidases have been identified, and most of them are isolated from fungi [16,17,18,19,20,21]. A typical example of a commercial α-galactosidase is that derived from *Aspergillus niger* mold, which is able to enact its function in the gastrointestinal tract by breaking down specific non-absorbable oligosaccharides before they are metabolized by colonic bacteria [15,17,18,19,22,23,24,25].

Increasing consumer awareness regarding the benefic impact of enzyme-containing dietary supplements has shifted industry commercial trends towards promoting their market expansion [8,14,19]. Dietary supplements and enzyme must be produced in accordance with Food and Drug Administration (FDA) regulations for Current Good Manufacturing Practice (cGMPs) to help and promote safe production and to facilitate transparency and uniformity in the industries [26,27,28,29]. The Food and Drug Administration regulates all aspects of dietary supplement in terms of quality, safety, labeling, and marketing [29,30]. For each component of an enzyme-based supplement an identity specification must be established, together with the limits of contamination and the stability profile under various conditions [26,27,28,29,30].

Dietary preparation efficacy can be verified by the testing of enzymatic activity [19,31,32]. The combination of moisture and temperature can cause the rapid deterioration of product integrity and enzyme activity levels. Although all enzymes lose potency depending on storage conditions, each enzyme may have a unique stability profile [31,32,33]. For this reason, stability tests are based on appropriate and validated protocols suitable for enzymes, especially those completed at the finished product stage. Specifications with regard to the stability of α-galactosidase as well as the evaluation of potency, integrity, and enzyme activity levels need to be defined and labeled by the dietary producer [31,32,33,34].

To ensure that a new process can produce reliable results, all laboratories should guarantee that the analytical method’s performance meets the requirements during all steps of the validation [31,35,36,37]. Assay corroboration provides an assurance of reliability during normal use and is referred to for documented evidence that the method does what it is intended to do [31,32,38,39,40]. For enzymatic activity, high levels of accuracy and reproducibility are recommended. A well-defined substrate with adequate lot-to-lot uniformity should be used [19,31,32]. Validation of enzyme assays should document assay specificity, sensitivity, variability, and assay linearity [26,27,29,35].

In this work, we proposed an intra-laboratory validation of an enzymatic assay for the quantification of α-galactosidase activity in commercial dietary supplements using a spectrophotometer measurement. Starting with methods indicated in literature, the Plackett–Burman statistic design was employed for the identification of the significant effects and the nominal levels of all factors involved in the measurement to achieve the best performance [36,37,38,39,40,41]. The highly selective enzymatic reaction design allowed for the quantification of α-Gal activity without any interference. Following the regulatory requirements of The International Council for Harmonisation of Technical Requirements for Pharmaceuticals (ICH), method validation was established, calculating the specificity, linearity, accuracy, intermediate-precision repeatability, and system precision of all obtained measurements [31,32,33,34,42]. To estimate the concordance and the linearity between the obtained results, an additional comparison with an alternative fluorimetric assay was performed. All these documented steps allowed for data traceability and avoided incorrect quantification, which could have unpleasant economic consequences for the laboratory producer.

## 2. Results

### 2.1. Plackett–Burman Test

The finished products were prepared and analyzed according to the procedure defined (see Section 4.3.3). The theoretical value of the enzyme, considered as reference, was estimated at 200 GalU/sachet, with specification criteria ≥170 GalU/2 g, as declared by the producer.

The Plackett–Burman test was conducted considering seven variables, including the dummy factors, in order to estimate the random measurement errors that occurred during enzyme activity quantification.

**Figure 1:**
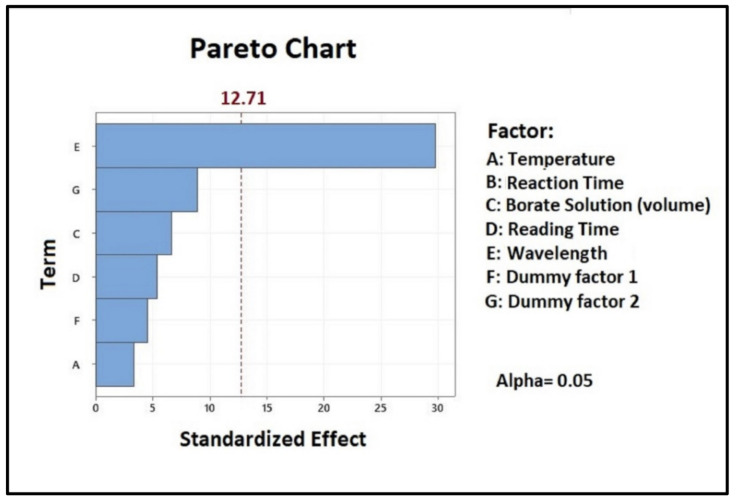
Pareto chart showing the effect of each variable examined during the assay set-up.

The design investigated every input factor involved during the analytical setting and arranged each of them in a Pareto chart (Figure 1). Based on the magnitude of its influence, each variable was expressed with a positive or negative sign, respectively. Only the wavelength parameter, represented by the blue bar exceeding the vertical red line, showed a significant influence on the α-Gal calculation, considering a 95% confidence level. All other variables considered, such as temperature, reaction time, borate solution volume, reading time, and dummy factors, showed no significance in α-galactosidase activity assessment and were kept constant in all further analyses (Tables S1–S3).

In order to estimate the *p*-values of each component, all raw data obtained during the enzymatic activity set-up were subjected to multiple linear regression analysis. In the linear regression coefficient determination, the adjusted R^2^ of 99.35% indicated that the model equation, given in uncoded units, was significant and could explain 99.35% of the variability in the response data. The equation revealed that all coefficients had a negative sign, so all factors showed a negative effect on the enzymatic assay. Furthermore, as already revealed by the Pareto chart, the only significant variable was the wavelength (*p* = 0.02). All the other factors were not statistically significant (*p*-value > 0.05 [43]).

To test the robustness of spectrophotometer method, three different samples were performed in triplicate, setting the wavelength at 420 nm. The average result (185.55 GalU/2 g) was compliant with the acceptance criteria (α-Gal specification of ≥170 GalU/2 g). The Standard Deviation (SD) 1.911 and residual standard deviation % (RSD%) 0.6419% demonstrated good reproducibility.

### 2.2. Validation Results

The enzymatic method was validated in the conditions established previously with the Plackett–Burman test, following both the ICH and the FDA guidelines. The specificity, linearity, accuracy, intermediate-precision, repeatability, and system precision of the method were defined.

#### 2.2.1. Specificity

The method’s specificity was evaluated by comparing the UV spectra obtained from the diluent, the placebo solution, the standard solution, and the finished product solution. For this purpose, the different absorbance values obtained were compared in order to define the background signal not derived from the enzyme supplement.

The absorbance values of both the placebo and blank solution were considered negligible (0.0075 and 0.0044, respectively), while the raw material and the finished product had the same absorption (0.5797).

The absorption peak of the α-galactosidase at 420 nm was unchanged in the presence of the other components of the commercial product formulation, demonstrating the specificity of the enzymatic method.

#### 2.2.2. Linearity

The linearity of our assay was considered as the ability of the method to have a linear response according to the increase or reduction in the active ingredient’s concentration.

The method’s linearity was defined by evaluating test proportionality with respect to the enzyme concentration within a specific range.

Spiked solutions were prepared using the following amounts of α-galactosidase enzyme: 50%, 70%, 100%, 130%, and 150% of theoretical concentration. For each concentration tested, three different dilutions were prepared from the mother solution. Each solution was analyzed individually.

The linear regression obtained at 420 nm could be expressed with the following equation:

y = 0.005 + 30.804x(1)

The *goodness-of-fit* (R^2^) was 0.9966, indicating a good linear relationship between the α-Gal concentration and the absorption peak.

#### 2.2.3. System Precision, Repeatability and Intermediate Precision

System precision, method repeatability, and intermediate precision were determined using several measurements of the finished product. System precision was established by measuring six readings of the same finished product solution at the concentration of 100% on the same day. The average result within the day was 182.7 GalU/2 g, and the obtained RSD% was 0.134%.

The analytical results obtained using the laboratory equipment used for this analytical setting should be considered as precise. Method precision was established by six assay determinations from the same batch of the finished food supplement product on the same day, while the intermediate precision was evaluated performing quantitative determinations on different days using different operators and reagents.

The results of method precision and intermediate precision are reported in Table 1.

Repeatability was measured with the same intra-day working conditions, while intermediate precision was measured on two different days (inter-day).

RSD values were below 5% for each single parameter, demonstrating that the method of α-Gal quantification had both excellent repeatability and intermediate precision.

The calculated Student’s *t*-value and F-value were found to be less than the tabulate values (considering five degrees of freedom) indicating no significant differences between the two analyses performed by two operators on different days.

#### 2.2.4. Accuracy

The accuracy of the presented enzymatic assay was evaluated as the ability of the method to provide an analytical response as close as possible to the real α-Gal value declared by the producer.

Accuracy. estimated at three different levels (80%. 100%. and 120%). reached a recovery rate of between 94% and 104%. The average recovery % of each single level was as follows: at 80% level the recovery rate was 99.57%; at 100% level the recovery was 97.89% while at 120% nominal level the recovery rate was 100.69%. The percentage of RSD for the low level was 4.3%, in the middle level was 3.17% and for the upper level was 2.57%. The values obtained for the accuracy evaluation were between 80% and 120% with respect to the real quantity of α-Gal spiked in test samples. never exceeding 10% of the expected concentration. These results indicated the reliable applicability of the method in routine food supplement and drug analysis.

### 2.3. Agreement between Methods

The comparison between two analytical methods allowed for an estimation of the degree of agreement of measurements. A method comparison allowed us to determine the quality of the results and validity of our assay for α-Gal activity quantification expressed in g/100 g using eight sachets belonging to three different batches. Results obtained by the optimized spectrophotometer method (method 1) were matched with those reached by a fluorimetric gold standard method (method 2) for human α-Gal quantification in biological samples. Raw data regarding 12 sachets from the product are reported in Table 2.

**Figure 2:**
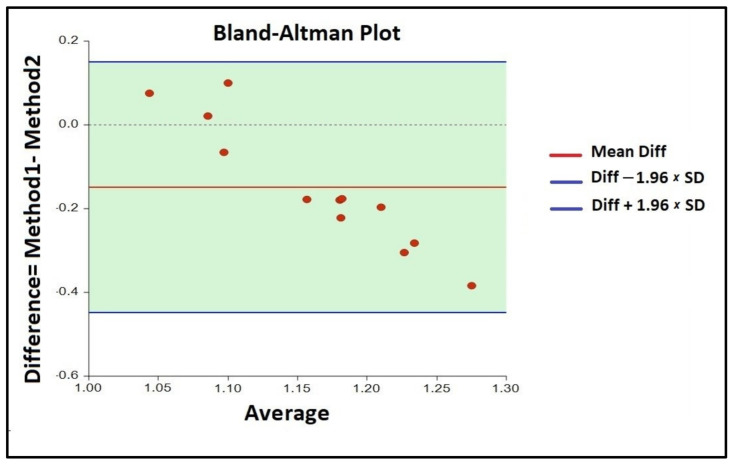
Bland–Altman plot for data from the Table 4, with the representation of the limits of agreement (blue line) from minus 1.96 × SD to + 1.96 × SD.

The Bland–Altman (B&A) plot reported in Figure 2 shows the average of the paired values from each method on the *x*-axis and the difference of each pair of readings on the *y*-axis.

In the B&A plot the average of two measurements was plotted along the horizontal axis, where the difference between the two methods was plotted along the vertical axis. All bias fell within the limits of agreement. No outliers of trend and patterns were present. Data presented in the plot were distributed with random variability. In the descriptive statistics presented in Table 3, the confidence intervals for the two variables and their difference were computed; while in the Bland–Altman analysis (Table 4) the bias and the upper and lower limit of agreement were calculated.

**Table 3:** Descriptive statistics.

Variable	Count	Mean	Standard Deviation	95.0% LCL of the Mean	95.0% UCL of the Mean
**Method 1**	12	1.09	0.02	1.07	1.10
**Method 2**	12	1.24	0.14	1.14	1.33
**Difference**	12	−0.15	0.15	−0.25	−0.05

**Table 4:** Bland–Altman analysis: Bias and limits of agreement for the two methods. Limits of agreement = diff ± 1.96 × (Std dev of difference) LCL: Lower Confidence Limit; ULC: Upper Confidence Limit.

Parameter	Count	Value	Standard Deviation	95.0% LCL of the Mean	95.0% UCL of the Mean
**Bias** **(Difference)**	12	−0.15	0.15	−0.25	−0.05
**Lower Limit of Agreement (LL)**	12	−0.45	0.08	−0.62	−0.28
**Upper Limit of Agreement (UL)**	12	0.15	0.08	−0.02	0.32
**Test of the normality of differences assumption:**					
**Assumption**	**Value**		**Prob. level**	**Decision (α = 0.050).**	
Shapiro–Wilk	0.937		0.4554	Cannot reject normality	

The Shapiro–Wilk test (Table 4) confirmed the normal distribution of the observations.

Taking into account all data reached, we demonstrated that the two methods could be interchangeably and equally applicable.

## 3. Discussion

The concentrations of active compounds in dietary supplements should be standardized to assure a reproducible and effective product for the consumer. Quality control and quality assurance procedures are needed to better define supplement ingredients and guarantee health outcomes [26,27,28,29].

In order to guarantee production integrity and efficacy to applicants, research laboratories are required either to follow certified guidelines or perform validation studies for their own developed procedures [31,32,34].

From this perspective, a rapid method for the measurement of α-galactosidase activity in food supplements is here proposed as a quality control indicator for industrial production.

α-Galactosidases are carbohydrates able to catalyze the hydrolysis of α-linkages in galacto-oligosaccharides such as raffinose, melibiose, stachyose, Verbascose, Galactomannans, and glycoconjugates. α-Galactosidase application in dietary supplements increases the intestinal digestion of α-galactosides by hydrolyzing the flatulence-causing sugars in processed food [1,2,3,4].

To guarantee high output, gut proteases should be avoided and highly efficient protease-resistant α-galactosidases are thus required in the food industry. However, only few α-galactosidases isolated from fungi have been identified with such properties thus far [15,17,18]. To provide the highest quantities of alpha-galattosidases. gut proteases should be excluded and highly efficient protease-resistant α-galactosidases are thus of great interest in the food industry. However, to date only a few α-galactosidases have been identified with such properties [15,17,18,19] and have mainly been isolated from fungi [22,23,24,25]. As most of alpha-galactosidases in the food industry are obtained from probiotic bacteria like bifido and lactic acid bacteria [16,17,19,21,22], and due to the fact that a large number of these are sensitive to gut proteases, the maximum activity of commercial α-galactosidases should be assured to the customer. To date, few studies have been carried out on α-galactosidase determination in dietary supplements [16,17,18,21,23,24]. Our investigations showed how to validate an assay for enzymatic activity evaluation using a simple spectrophotometric measurement at 420 nm. We employed eight sachets belonging to the same batch of commercial dietary supplements containing ≥170 GalU of α-galactosidase per sachet. The Plackett–Burman statistic design was initially used to study all variables involved in the analytical setting. Among several variables taken into consideration, the wavelength represented the only significant one. Temperature, reaction time, borate solution volume, reading time, and dummy factors had no significance in α-galactosidase activity assessment and were kept constant during the validation analysis. The choice of correct statistical procedures for data exploration and the interpretation of results are important keys for the proper assessment of method trueness. Assay optimization and the pre-validation step are important to determine how a range of matrix and sample elements, as well as assay conditions, can affect assay performance [35,36,37,38,39,43,44,45].

To test the robustness of the new method, several serial measurements at constant conditions were performed. All results were in agreement with the quantities declared by the suppliers (≥170 GalU/sachet). Comprehensive experiments to evaluate and report the quantitative performance of the presented assay, including specificity, linearity, accuracy, intermediate-precision, repeatability and system precision, were carried out following both ICH and FDA guidelines [26,27,28,29,30]. The unique absorption peak obtained at 420 nm by α-Gal enzyme was not influenced by other excipients contained in the commercial product formulation, demonstrating a 100% specificity of the assay.

The linearity of our assay was considered as the ability of the method to have a linear response according to both an increase and a reduction in active ingredient concentrations. The method’s linearity was defined as the ability of the assay to return values that were directly proportional to the concentration of the target analyte spiked in the sample. Spiked solutions with 50%, 70%, 100%, 130% and 150% of theoretical concentrations of α-galactosidase in placebo were prepared and analyzed. No significant deviations were found with respect to the expected amount of enzyme. System precision was determined by replicating six determinations of the same finished product solution at a concentration of 100% under normal assay conditions. As expected for enzymatic assays, precision was <10% [31,32,34].

Precision included the assay´s within-day repeatability and day-to-day reproducibility by different operators and with different reagents. The RSD% of the system precision was 0.134%, considering a cut-off value of 5.0%. The analytical results reached were considered very precise. The accuracy of the presented analytical assay was estimated at three different nominal levels: 80%, 100% and 120%. The recovery rate ranged between 94% and 104%. Taken together, these results confirmed the strength of our method for quality control.

By comparing our method with a reference gold standard assay for α-galactosidase quantification we looked for a potential measurement bias. The comparison between the two methods was performed on eight sachets from three different batches. The results confirmed that the introduction of the new method would not affect the quality of analytical control assessment of the finished dietary product.

## 4. Materials and Methods

### 4.1. Commercial Supplement Composition

The composition of the commercial dietary supplement subject to analysis included excipients and other ingredients such as fructose, fructo-oligosaccharides, a mix of enzymes, botanical dry extracts and flavors. The specification of enzymatic activity contained in each sachet was ≥170 GalU/sachet (theoretical value at 100% = 200 GalU/sachet, where the sachet weight was 2 g). In particular, 8 sachets from 2 batches were employed for the strategy planning by the Plackett–Burman test; 6 sachets from a single batch were used for all validation procedure (specificity, linearity and accuracy determination) and 8 samples belonging to 3 different batches were tested in comparison with the gold standard fluorimetric method.

During the analysis, the content of excipients and other ingredients was not taken into consideration. Only measurements of the global activity using placebo (dietary supplement without α-galactosidase enzyme) were taken in order to underline any possible background interference or unspecific signal detection.

### 4.2. Design of Experiment: Strategy Planning

In order to study the simultaneous variation of the factors on the considered responses, a multivariate approach using an experimental design by Plackett–Burman was employed [35,36,39]. A fractional factorial Plackett–Burman design was used to screen and evaluate the significant variables that could influence the enzymatic assay method, because this model does not explain the interaction among various variables [36,37,39,44].

The Plackett–Burman statistical design is very frequently used to study the effects of the analytical determination. It is a 2-factor (i.e. −1 and +1) design that locates significant variables for the production by screening “n” variables in “n + 1” experiments. All 7 factors chosen in the present investigation were tested at these 2 levels based on the Plackett–Burman matrix design. The main effect was calculated basically as a difference between the average measurements of each variable made at a high level (+1) and a low level (−1) (Table 5). This design screened variables based on a first-order model:

Y = β_0_ + ∑β_i_X_i_ (2)

where Y was the response (α-galactosidase activity), β_0_ was the model intercept, and β_i_ was the variable estimates. In this study, the variables were screened using Minitab version 19.2020.1 software.

The experiments were carried out according to the matrix shown in Table 5, where each row represents 1 trial while each column represents a single variable. Two dummy variables were studied to calculate the standard error. The results of experiments were obtained applying the spectrophotometric approach described in Section 4.3.

### 4.3. Spectrophotometry

#### 4.3.1. Chemicals and Materials

The reagents and chemicals used for the spectrophotometer quantification were: sodium borate decahydrate (*Carlo Erba)*, sodium hydroxide (*Carlo Erba*), sodium acetate (*Carlo Erba*) and acetic acid (*Chem Lab*). For the preparation of *acetate buffer*, 3.49 g of sodium acetate was diluted in 800 mL of purified water with 0.4 mL of acetic acid then added and taken to a final volume of 1000 mL with purified water. The optimal pH value was 5.5 ± 0.1. The *substrate solution* was composed of 105 mg of 4-nitrophenyl-α D galacto-pyranoside (*Sigma Aldrich*) dissolved into 50 mL of acetate buffer solution. The last solution needed for the enzymatic activity was *borate solution* composed of 23.8 g of sodium borate decahydrate in 1000 mL of purified water, where the pH was corrected at 9.7 ± 0.1.

The UV–Visible spectrophotometer (*JASCO*) employed for analysis was initially qualified and calibrated in accordance with cGMP before this activity was conducted.

#### 4.3.2. Preparation of Standard Solutions

For standard solution preparation 0.130 g of α-galactosidase working standard was added to 50 mL of purified water and agitated magnetically until complete dissolution (*sol std M*). Then. 0.1 mL of the *sol std M* was added to 100 mL of purified water *(sol std 1).* For *standard solution 2* (*sol std 2)*, 0.2 mL of the *sol std M* was diluted in 100 mL of purified water. For *standard solution* 3 (*sol std 3*), 0.35 mL of the *sol std M* was diluted in 100 mL of purified water.

The calibration curve was constructed analyzing 3 different concentrations of the standard solutions (sol std 1—sol std 2—sol std 3) on the same day.

For blank sample, 1 mL of purified water was included for measurement correction.

Spiked samples composed of mixtures of placebo in suitable proportions and the finished products were prepared in order to evaluate enzymatic activity.

#### 4.3.3. Procedure for Alpha-Galactosidase Quantification

One milliliter of standard or sample solution was incubated with 2 mL of substrate solution at 37 °C for 15 min. in the dark. To stop the reaction, 5 mL of borate solution was added to each sample. As usual, substrate solution (no enzyme), blank (only water) and enzyme blanks (enzyme but no substrate) were included for correction. Standards and samples were immediately read in the UV–Visible spectrophotometer. Assays were performed in triplicate. Procedure steps are summarized in Table 6 below:

The quantification of total α-galactosidase activity was calculated from the following equation:

(3) $$\frac{{C\;\times \;V}_{f}}{{\mathrm{Pcp}\;\times \;V}_{I}}\;\times \;2g=\mathrm{Alpha}\;\mathrm{galactosidase}\;\left(\frac{\mathrm{GalU}}{\mathrm{sachet}}\right)$$

where
C=Concentration of the alpha-galactosidase (GalU/mL) in the sample solution obtained by interpolation of straight calibrationPcp=Weight of sample (g)2g=Weight of the sachet (2 g)V_I_=Volume of sample pour in the last dilutionV_f_=Final volume dilution (mL)

### 4.4. Fluorimetric Alpha-Galalactosidase Assay

To assess the performance of the newly developed method, a comparison against a standard method for human α-galactosidase quantification by fluorimeter was conducted. α-Galactosidase activity was measured by a fluorimetric enzyme assay on 12 sachets from the same batch. The assay mix included 50 μL of raw material corresponding to 500 ng of 4-methylumbelliferyl-α-D-galactopyranoside as a substrate (Sigma Chemical; final concentration of 6.7 mM) and sodium acetate buffer (pH 4.5; final concentration 0.13 M) in a final volume of 300 μL. The reaction mix was incubated for 30 min at 37 °C in the dark. To stop the reaction, 1700 µL of buffer carbonate (0.5 M pH 10.7) was added. The assay was repeated with and without the inclusion of N-acetylgalactosamine (GalNAc) at a 100-mM final concentration in the previous assay mixture. GalNAc is an inhibitor of the lysosomal enzyme α-N-acetylgalactosaminidase. also known as α-galactosidase B because in vitro it has some nonspecific activity toward the artificial substrate used for assay of α-galactosidase B. In this case, substrate solution (no enzyme), blank (only water) and enzyme blanks (enzyme but no substrate) were included for background and non-specific corrections.

Readings were performed to measure fluorescence intensity (Excitation/Emission= 360/445 nm) at room temperature using an end-point setting. A calibration curve was obtained by linear regression of the absorbance readings versus concentration using 10 different solutions with known concentrations of the analyte.

### 4.5. Method Comparisons

The method comparisons assessed the degree of agreement between the developed enzymatic method and the second fluorimetric method. The aim of the method comparisons was to investigate the measurement discrepancies between the 2 different analytical methods. Bland and Altman introduced the Bland–Altman plot (B&A) to describe the agreement between quantitative measurements [45]. By using a graphical approach, the limit of agreement between two quantitative measurements could be calculated. The resulting graph is a scatter plot (*xy*), in which the *y-*axis shows the difference between the two paired measurements (A − B) and the *x*-axis represents the average of these measures ((A + B)/2). In other words, the difference of two paired measurements is plotted against the mean of the two measurements. The lack of agreement was evaluated by calculating the bias, estimated by mean difference (d) and the standard deviation (s). B&A considered that most differences would lie between d − 2s and d + 2s or more precisely, that 95% of the differences will be between d − 1.96s and d + 1.96s if the differences are normally distributed (Gaussian). The normal distribution of the differences was verified using the Shapiro–Wilk test with NCSS statistical software (NCSS 2020. v20.0.2).

## 5. Conclusions

In the present work a rapid method was developed and validated for the routine determination of α-galactosidase in food supplements. The specificity, linearity range, precision, accuracy and repeatability proved to be suitable for the enzyme quantification of commercial dietary preparations. The specificity was 100% and the method precision was less than 10% with an RSD% of 0.134%. while the accuracy ranged from 94% to 105% with respect to the theoretical concentration spiked in the placebo. Sample recoveries were in good agreement with the respective theoretical declaration. According to FDA and ICH guidelines the quantitative performances were all statistically significant.

All the experiments conducted to evaluate specificity, linearity, accuracy, intermediate-precision, repeatability and system precision were carried out in technical compliance with the acceptance limits described by the main regulatory organizations.

No significant differences were found between our spectrophotometric method and the reference method.

The proposed method can be used for the routine quality control analysis of food supplement preparations containing α-galactosidase.

The product validation design presented here could integrate statistical model-based and experimental-based techniques. This “bi-modal” approach could represent a challenge in quality control assessment and innovative and more sustainable processing routes for the active chemical and biological ingredients of food supplements.

## Figures and Tables

**Table 1:** The raw data obtained and employed for the determination of method precision and intermediate precision. For each calculation the average, the residual standard deviation (RSD), the calculated Student’s *t*-value (t), and F-values (F)were computed RSD: Relative Standard Deviation, DS: Standard Deviation.

Analyst 1 (First Day)			Analyst 2 (Second Day)		
SAMPLE	Sample Weight (g)	Content Found (GalU/sachet)	Sample	Sample Weight (g)	Content Found (GalU/sachet)
A_1_	2.098	181.93	B_1_	2.149	185.95
A_2_	1.974	195.52	B_2_	2.068	181.53
A_3_	2.098	182.63	B_3_	2.056	189.01
A_4_	2.094	185.41	B_4_	2.036	186.35
A_5_	2.091	184.14	B_5_	2.120	180.94
A_6_	2.117	185.79	B_6_	2.029	183.83
**AVERAGE**		185.91	**AVERAGE**		186.40
**DS**		4.95	**DS**		3.089
**RSD%**		2.661%	**RSD%**		1.673%
**RSD% TOTAL**					2.154%
*F* calculated	2.57		Ftabulate		5.05
*t* calculated	0.550		*t* tabulate		2.228

**Table 2:** Results comparison between the 2 analytical methods.

Spectrophotometer (Method 1)	Fluorimeter (Method 2)
g/100 g	
1.1501	1.0500
1.0743	1.3790
1.0907	1.2700
1.0832	1.4670
1.0929	1.3750
1.0938	1.2700
1.0678	1.2456
1.1118	1.3082
1.0962	1.0749
1.0644	1.1298
1.0814	1.0057
1.0702	1.2920

**Table 5:** Plackett–Burman screening design of experiments and their results. X1: temperature (−1: 35 °C. 1: 39 °C); X2: Reaction Time. (−1: 13 min. 1: 18 min); X3: borate solution volume (−1: 3 mL. 1: 7 mL); X4: reading time (−1: 20 min. 1: 45 min); X5: wavelength (−1: 390 nm. 1: 420 nm); X6 and X7: dummy factors. The responses reflect the enzymatic activity obtained (Y) expressed in GalU/2 g**.**

ID	X1	X2	X3	X4	X5	X6	X7	Y
**1**	1	1	1	−1	1	−1	−1	60.50
**2**	−1	1	1	1	−1	1	−1	149.10
**3**	−1	−1	1	1	1	−1	1	14.84
**4**	1	−1	−1	1	1	1	−1	44.30
**5**	−1	1	−1	−1	1	1	1	47.20
**6**	1	−1	1	−1	−1	1	1	118.74
**7**	1	1	−1	1	−1	−1	1	144.86
**8**	−1	−1	−1	−1	−1	−1	−1	208.50

**Table 6:** Summary of the assay’s workflow preparation.

Time (Minutes)	Reagent	Blank	Sample	Standard	Reference
**T = 0**	Substrate solution	2 mL	2 mL	2 mL	2 mL
**T = 5**	Borate solution	/	/	/	5 mL
	α-Galactosidase	/	/	1mL	/
	H_2_O	1 mL	/	/	/
	Sample	/	1 mL	/	/
**T = 20**	Borate solution	3 mL	3 mL	3 mL	/
	Sample/standard	/	/	/	1 mL

## Data Availability

The data presented in this study are available on request from the corresponding author.

## Supplementary Materials

The following are available online. Table S1: Estimated effects and relative coefficient of each variable tested in the Plackett-Burman design; Table S2: Summary of Plackett-Burmann test model; Table S3: Variance Analysis in Plackett-Burmann test.

## Sample Availability

Samples of the compounds are available from the corresponding author.

## Abbreviations

α-Gal	Alpha-galactosidase
cGMP	Current Good Manufacturing Practice
FDA	Food and Drug Administration
GalNAc	N-acetylgalactosamine
GalU	Galactosidase units
ICH	International Council for Harmonization of Technical Requirements for Pharmaceuticals
RSD	Relative Standard Deviation
DS	Standard Deviation
