# Supplementary material for: Intra-Laboratory Validation of Alpha-Galactosidase Activity Measurement in Dietary Supplements
Source: Molecules. 2021 Mar 12;26(6):1566. doi: 10.3390/molecules26061566 (PMC8000382; doi:10.3390/molecules26061566)
Supplement: Supplementary file 1 [file molecules-26-01566-s001.pdf]

**Table S1.** Estimated effects and relative coefficient of each variable tested in the Plackett-Burman design.

| Term | Effect | Coef | SE Coef | *t*-Value | *p*-Value | VIF |
|---|---|---|---|---|---|---|
| Constant | | 98.51 | 1.91 | 51.57 | 0.012 | |
| Temperature | -12.81 | -6.40 | 1.91 | -3.35 | 0.184 | 1.00 |
| Borate solution (volume) | -25.42 | -12.71 | 1.91 | -6.65 | 0.095 | 1.00 |
| Reading time | -20.46 | -10.23 | 1.91 | -5.36 | 0.118 | 1.00 |
| Wavelenght | -113.59 | -56.79 | 1.91 | -29.74 | 0.021 | 1.00 |
| Dummy 1 | -17.34 | -8.67 | 1.91 | -4.54 | 0.138 | 1.00 |
| Dummy2 | -34.19 | -17.09 | 1.91 | -8.95 | 0.071 | 1.00 |

**Table S2.** Summary of Plackett-Burman test model.

| S | R-sq | R-sq (adj) | R-sq (pred) |
|---|---|---|---|
| 5.40230 | 99.91% | 99.35% | 94.02% |

**Table S3.** Variance Analysis in Plackett-Burman test.

| Source | DF | Adj SS | Adj MS | *f*-Value | *p*-Value |
|---|---|---|---|---|---|
| Model | 6 | 31202.4 | 5200.4 | 178.19 | 0.057 |
| Linear | 6 | 31202.4 | 5200.4 | 178.19 | 0.057 |
| Temperature | 1 | 328.2 | 328.2 | 11.25 | 0.184 |
| Borate solution (volume) | 1 | 1292.4 | 1292.4 | 44.28 | 0.095 |
| Reading time | 1 | 837.2 | 837.2 | 28.69 | 0.118 |
| Wavelenght | 1 | 25805.4 | 25805.4 | 884.21 | 0.021 |
| Dummy 1 | 1 | 601.4 | 601.4 | 20.60 | 0.138 |
| Dummy2 | 1 | 2337.9 | 2337.9 | 80.11 | 0.071 |
| Error | 1 | 29.2 | 29.2 | | |
| Total | 7 | 31231.6 | | | |
